# Fabrication of high performance based deep-blue OLED with benzodioxin-6-amine-styryl-triphenylamine and carbazole hosts as electroluminescent materials

**DOI:** 10.1038/s41598-023-50867-x

**Published:** 2024-01-29

**Authors:** E. Dhineshkumar, N. Arumugam, E. Manikandan, M. Maaza, Abhishek Mandal

**Affiliations:** 1 Manushyaa Blossom Pvt. Ltd., Chennai, Tamil Nadu 600102 India; 2 Department of Biotechnology, School of Life Sciences, Pondicherry University (A Central University), Puducherry, 605014 India; 3 Centre for Nanoscience and Technology, MSGET, Pondicherry University (A Central University), Puducherry, 605014 India; 4 College of Graduate Studies, UNESCO-UNISA Africa Chair in Nanosciences-Nanotechnology, Muckleneuk Ridge, PO Box 392, Pretoria, South Africa; 5 Nanosciences African Network (NANOAFNET), iThemba LABS-National Research Foundation, 1 Old Faure Road, Somerset West, PO Box 722, Western Cape, 7129 South Africa

**Keywords:** Chemistry, Materials science, Nanoscience and technology, Optics and photonics, Physics

## Abstract

The present study reports synthesis of phenathroimidazole derivatives structures following donor–acceptor relation for high performance deep-blue light emitting diodes. Herein, methyl substituted benzodioxin-6-amine phenanthroimidazoles Cz-SBDPI and TPA-SBDPI derivatives that provide the blue light were designed and synthesized. These Cz-SBDPI and TPA-SBDPI show higher glass transition (T_g_) temperatures of 199 and 194 °C and demonstrate enhanced thermal properties. Apart from enhanced thermal stability these compounds also exhibit superior photophysical, electrochemical and electroluminescent properties. The non-doped carbazole based device display improved electroluminescent performances than those of TPA-based devices. The strong orbital-coupling due to decreased energy barrier between Cz-SBDPI transitions result in deep blue emission with CIE—0.15, 0.06. For non-doped Cz-SBDPI device; high L (brightness):12,984 cd/m^2^; η_c_ (current efficiency): 5.9 cd/A; η_p_ (power efficiency): 5.7 lm/W and η_ex_ (external quantum efficiency): 6.2% was observed. The results show that the D–A emitters can serve as simple but also as an effective approach to devise cheap electroluminescent materials that has high efficiency and can serve as OLED devices.

## Introduction

Recently, organic based light emitting diodes (OLEDs) have generated significant interest as they have lot of potential in optoelectronics applications. However, the major drawback associated with the fabrication procedure of these blue OLEDs is their large band gap (E_g_) and also the improper carrier injection^1–5^. Consequently, this high-energy gap effects in low electron affinities of these blue-emitters which hamper carrier injection/balances and thereby reduces the efficiency. The poor efficiencies of blue component results in white OLEDs. The emitter that experiences intermolecular or intramolecular interactions has aggregated arrangements that lead to bathochromic shift and reduced quantum efficiency^6^. So, in these fluorescent materials such as OLEDs, efficiency (η_s_) is only about 25% for these radiative decay process. Here, singlet excitons only are spin allowed and so return to their ground state. Thus, priority is given to research or studies that can develop highly efficient blue emitter with color purity^7,8^. In this direction, donor–acceptor (D–A) structures that contain bulky moiety provides steric hindrance have been used to reduce the quenching process^9–15^. This type of structural modification results in desired properties such as improved color purity, enhanced thermal stabilities and quantum efficiencies, bipolar properties in addition to higher exciton utilizing efficiency^16–20^. The methodology used in designing this kind of molecular structures has prospects in generating more efficient and cost-effective fluorescent based OLED materials with maximum electroluminescence (EL) efficiency that are comparable with that of other phosphorescent based materials. Taking all the above mentioned points into consideration, the present investigation aims at designing donor–acceptor derivatives: (1) Cz-SBDPI and (2) TPA-SBDPI from triphenylamine (TPA) and carbazole (Cz) that act as strong and weak donors, respectively. These materials comprises of moieties that are involved in hole (donor) and electron (acceptor) transport and is expected to show high quantum yields in films. The low value of singlet–triplet (∆_ST_) splitting suggests that the energy available is sufficient only to excite the triplet state (E_T_) of blue phosphorescent dopant. Therefore, this study focuses on synthesizing these derivatives that show balanced injection/transport abilities and impart high thermal stabilities required for the fabrication of high performance electroluminescent materials used as OLED devices.

## Experimental section

### Methods and spectral instrumentation

For thermal analysis, both DSC and TGA studies were conducted in N_2_ atmosphere on SEIKO instrument (Model No. SSC5200) where heating rate of 10 °C/min was employed. Absorption spectra for both solution and films were monitored via UV–Vis spectrophotometer (Perkin-Elmer Lambda 35). Time-resolved fluorescence decay was monitored with Horiba Fluorocube-01-NL (Source: Nano LED; Detector: TBX-PS) and decay analysis was carried out by employing time correlated single-photon counting (TCSPC) method with DAS6 software to determine the Fluorescence lifetime for these emissive materials. Moreover, the validity and evaluation of data fitting was confirmed from the reduced χ^2^ values. The fluorescence spectrometer (Model-F7100) was used to obtain quantum yield (PLQY).

#### Computational details

The parameters used for geometrical optimization involve ground state (DFT)/excited states (TD-DFT) carried out using Gaussian-09 Software^20^. Computational studies were performed to theoretically calculate the Energy for Oxidation (E_ox_) and also HOMO energy computed by the formula: E_HOMO_ = − (E_ox_ + 4.8 eV). Also, LUMO energy was calculated by deducting the band gap from HOMO values by the following equation, shown below: E_LUMO_ = E_HOMO_ − 1239/λ_onset_.

#### Fabrication of device and measurement

Fabrication of OLEDs was successfully carried out by sequential deposition of layers in the following order: NPB, Cz or TPA, TPBI, Lithium Fluoride (LiF) and the Al electrode over the substrate, i.e., the ITO glass. Subsequently, these organic materials were then thermally evaporated with ULVAC Cryogenics maintained at 0.14 mPa. Electroluminescence behaviour based on current–density–voltage changes in addition to CIE (x, y) coordinates were concurrently determined by Keithley 2450 m. The prepared compounds were additionally purified either via recrystallization or with silica based column chromatography. Further purification process is carried out by sublimation performed under vacuum (10^–6^ Torr) conditions. EL spectra and CIE coordinates was measured with USB-650-VIS–NIR spectrometer (Ocean Optics, Inc, USA).

#### Synthesis of 2-(4-bromostyryl)-1-(1-(2,3-dihydrobenzo[b][1,4]dioxin-6-yl)ethyl)-1H-phenanthro[9,10-d]imidazole (SDBPI)

**Figure 1:**
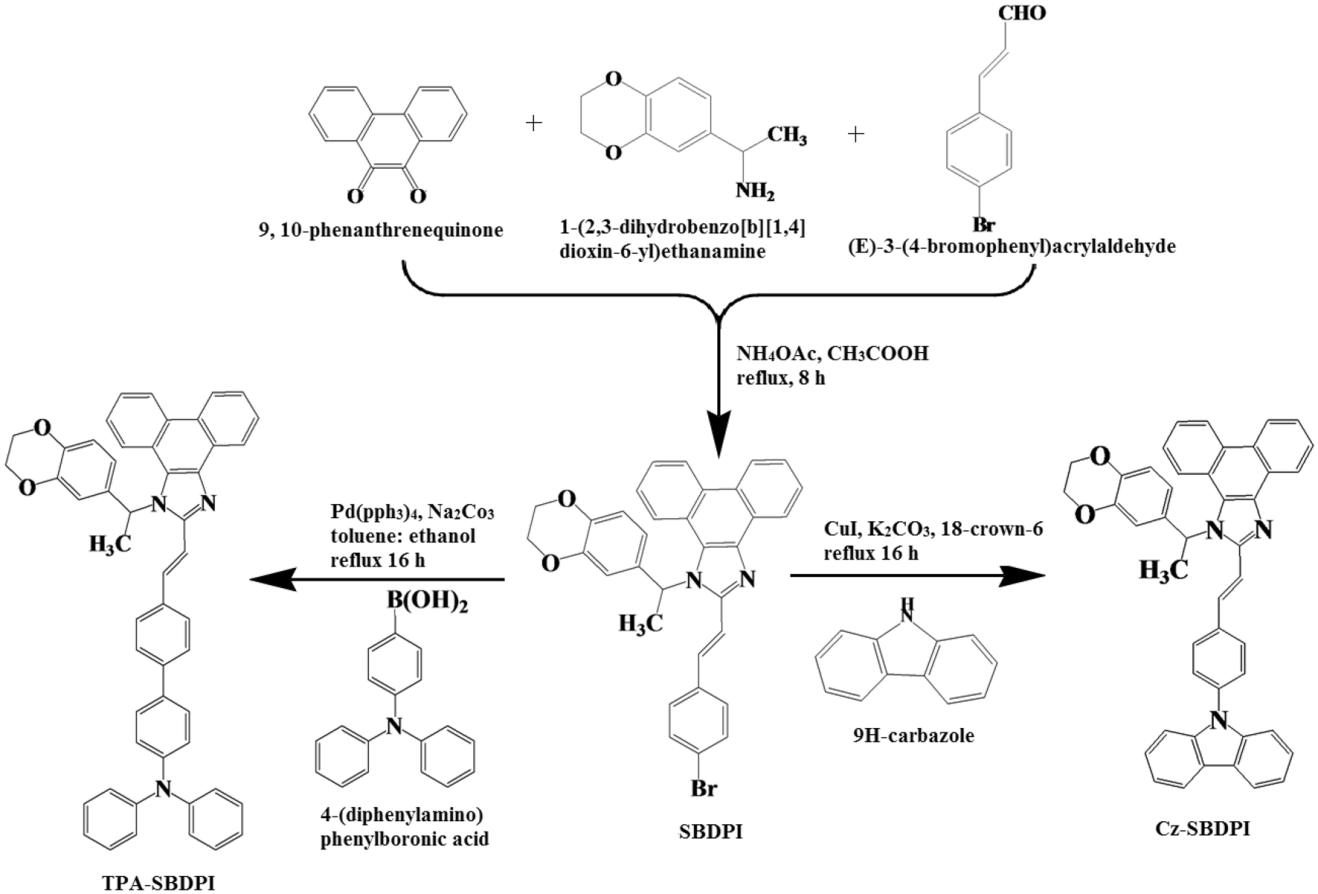
Synthetic route of SBDPI, Cz-SBDPI and TPA-SBDPI.

9,10-Phenanthrenequinone (2.07 g, 10 mmol), 3-(4-bromophenyl)acrylaldehyde (10 mmol), 1-(2,3-dihydrobenzo[b][1,4]dioxin-6-yl)ethanamine (6 mmol) and ammonium acetate (61 mmol) in acetic acid (25 mL) for 8 h under nitrogen atmosphere. The solvent was distilled off and the pure 2-(4-bromostyryl)-1-(1-(2,3-dihydrobenzo[b][1,4]dioxin-6-yl)ethyl)-1H-phenanthro[9,10-d] imidazole was separated out (see Fig. 1). Yield 66%. M. P. 246 °C. Anal. calcd. for C_37_H_25_BrN_2_O_2_.

#### Synthesis of 2-(4-(9H-carbazol-9-yl)styryl)-1-(1-(2,3-dihydrobenzo[b][1,4]dioxin-6-yl)ethyl)-1H-phenanthro[9,10-d]imidazole(Cz-SBDPI)

9,10-Phenanthrenequinone (5 mmol), 4-(9H-carbazol-9-yl)benzaldehyde (0.698 g, 2 mmol), and 2-(4-bromostyryl)-1-(1-(2,3-dihydrobenzo[b][1,4]dioxin-6-yl)ethyl)-1H-phenanthro[9,10-] imidazole, in CuI (0.05 mmol), 18-crown-6 (0.05 mmol) and K_2_CO_3_ (6.0 mmol) in tertrahydro-1,3-dimethylpyrimidin-2(1H)-one (2.0 mL) for 16 h under N_2_ stream. The solvent was distilled off and the pure Cz-SBDPI was separated out. Yield 58%. M.P. 234 °C. Anal. calcd. for C_45_H_33_N_3_O_2_.

#### Synthesis of 2-(4-(9H-carbazol-9-yl)styryl)-1-(1-(2,3-dihydrobenzo[b][1,4]dioxin-6-yl)ethyl)-1H-phenanthro[9,10-d]imidazole (TPA)

9,10-Phenanthrenequinone (5 mmol), 4-(diphenylamino)phenylboronic acid (7.5 mmol), 2-(4-bromostyryl)-1-(1-(2,3-dihydrobenzo[b][1,4]dioxin-6-yl)ethyl)-1H-phenanthro[9,10-]imidazole Pd(PPh_3_)_4_ (0.25 mmol) and aqueous Na_2_CO_3_ (15 mL) in toluene:ethanol (20:15 mL) for 16 h under N_2_ stream and the pure TPA-SBDPI Yield 60%. M.P. 230 °C. Anal. calcd. for C_51_H_39_N_3_O_2_.

### Procedure for device fabrication

OLEDs: ITO is employed as an anode, LiF/Al is used as a cathode, NPB is used as a hole transport layer (HTL), TCTA is used as a hole transport layer (HTL), as well as an electron blocking layer (EBL), and Cz-SBDPI/TPA-SBDPI is used as an electron transport layer (ETL). Configuration of deep blue-emitting devices of ITO/(N,N0-di-mtolyl-N,N0-diphenyl-1,10-biphenyl-4,40-diamine) TPD/(4,40-bis[N-(1-naphthyl)-N-phenylamino]biphenyl)NPB/(4,40,400-tris(N-carbazolyl) triphenylamine) TCTA (hole-transporting layer) HTL (50 nm)/Cz-SBDPI/TPA-SBDPI (Emissive layers) (30 nm)/(2,9-dimethyl-4,7-diphenyl-1,10-phenanthroline) BCP (15 nm)/(tris(8-hydroxyquinolinato) aluminium) Alq3 (50 nm)/(1 nm) LiF/(100 nm) was achieved by coating the pre cleaned ITO glass substrates at a resistance of 20 Ω/sq, over the Al electrode.

## Results and discussion

Here, the different materials used as blue emitters; 2-(4-(9H-carbazol-9-yl)phenyl)-1-(4-((E)-2-(1-(2,3-dihydrobenzo[b][1,4]dioxin-8-yl)-1H-phenanthro[9,10-d]imidazol-2-)vinyl) phenyl)-1H-phenanthro[9,10-d] imidazole (Cz-SBDPI) and 2-(4-(9H-carbazol-9-yl)phenyl)-1-(4-((E)-2-(1-((2,3-dihydrobenzo[b][1,4]dioxin-6-yl)methyl)-1H-phenanthro[9,10-d]imidazol-2-yl)vinyl) phenyl)-1H-phenanthro [9,10-d]imidazole (TPA-SBDPI) were synthesized having yields 50 and 58%, respectively. The route followed to devise these emissive materials is depicted as Fig. 1. The high photoluminescence efficiency (*η*_*PL*_) observed is ascribed to weak electron donating nature of the carbazole in Cz-SBDPI which results in the raise of % LE components in the emissive state (HLCT).

### Thermal analysis

**Figure 2:**
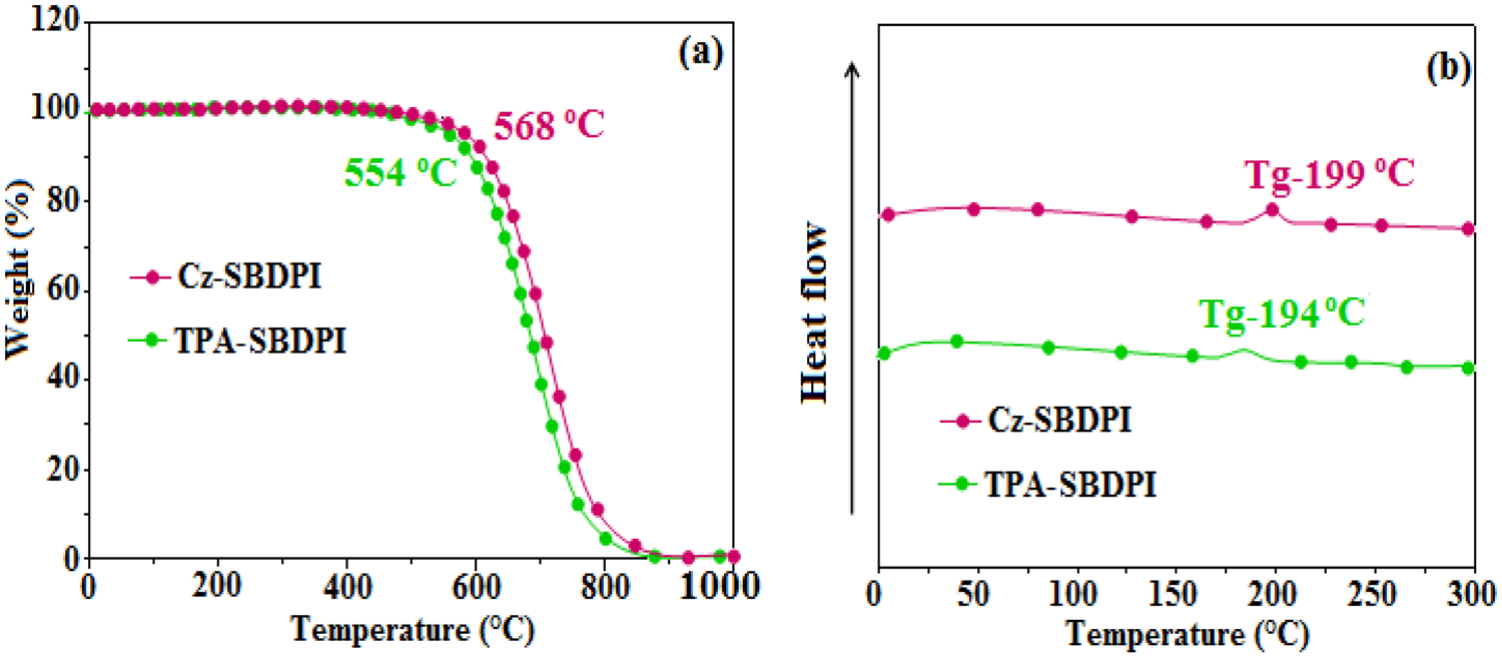
(**a**) TGA slope and, (**b**) DSC graphs of Cz-SBDPI and TPA-SBDPI phenanthrimidazoles.

Thermal based properties of prepared electrolumFinescent materials was conducted using TG and DSC analysis. The fabricated materials exhibited superior thermal stabilities courtesy to the inclusion of bulky moiety at imidazole carbon that renders it highly rigid. Moreover, the capping effect noticed at side chains of imidazole results in increase in the size of nitrogen atom that provides enhanced thermal stability (*T*_*d*_ and *T*_*g*_) so that the devices can work efficiently (Table 1). Thus, increase in *T*_*g*_ support amorphous nature and stable film formation via thermal evaporation, vital for OLEDs applications. It is also evident that Cz-SBDPI and TPA-SBDPI in particular demonstrate higher decomposition temperatures (*T*_d_) of 568 and 554 °C, respectively. Similarly, glass transition temperatures (*T*_g_) of 199 °C for Cz-SBDPI compared to 194 °C for TPA-SBDPI was also noticed (see Fig. 2). The enhancement in thermal stability associated with Cz-SBDPI in comparison with TPA is attributed to the increased rigidity noticed in the case of Cz-SBDPI useful as OLED. Moreover, higher Tg values also indicate that the stronger intermolecular forces in bond formations for C–N⋯H as well as C–N⋯Ph interactions because of amine groups and moieties present in phenanthrimidazole plane results in close confinement and tighter packing leading to higher rigidity^21^.

**Table 1:** The optical and thermal properties of Cz-SBDPI and TPA-SBDPI based devices.

Emitters	Cz-SBDPI	TPA-SBDPI
λ_ab_ (nm) (sol/film)	262,330/333,374	253,334/336,372
λ_em_ (nm) (sol/film)	410/426	414/416
T_*g*_/T_*d*_ (°C)	199/568	194/554
ɸ	0.80	0.75
HOMO/LUMO (eV)	− 5.40/− 2.65	− 5.45/− 2.60

**Figure 3:**
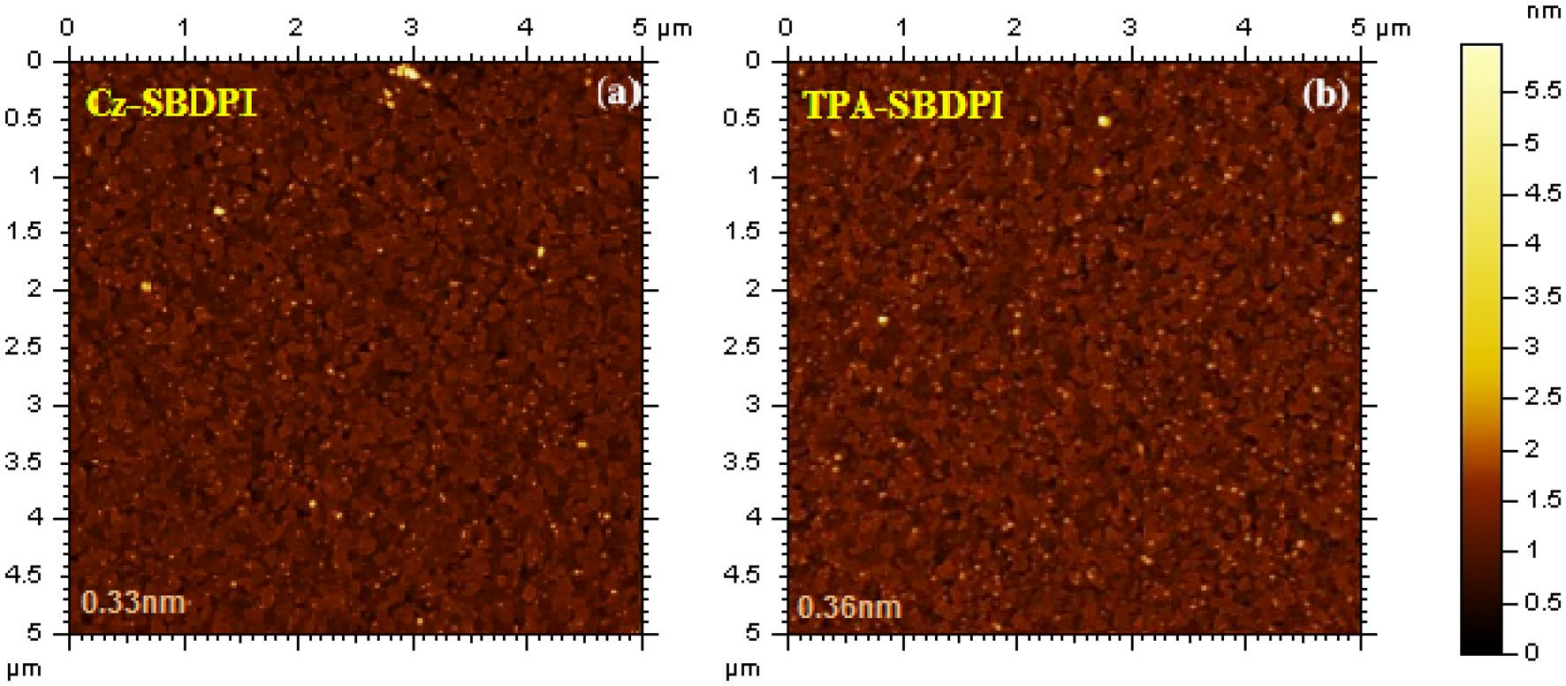
AFM images captured for Cz-SBDPI (**a**) and TPA-SBDPI (**b**) at 40 and 90 °C.

The stability of Cz-SBDPI and TPA-SBDPI thin films at different temperatures, 40–90 °C for 12 h was examined by atomic force microscopic (AFM) technique. The RMS of 0.33 nm (Cz-SBDPI) and 0.36 nm was noted for TPA-SBDPI. The thin-film surface depicts that no significant morphological changes occur prior to and later on annealing at 90 °C for 10 h which indicate that prepared samples are stable with respect to the variation in temperatures and thus confirms that the fabricated emissive materials are appropriate for use as OLED devices (Fig. 3).

### Photo-physical properties

**Figure 4:**
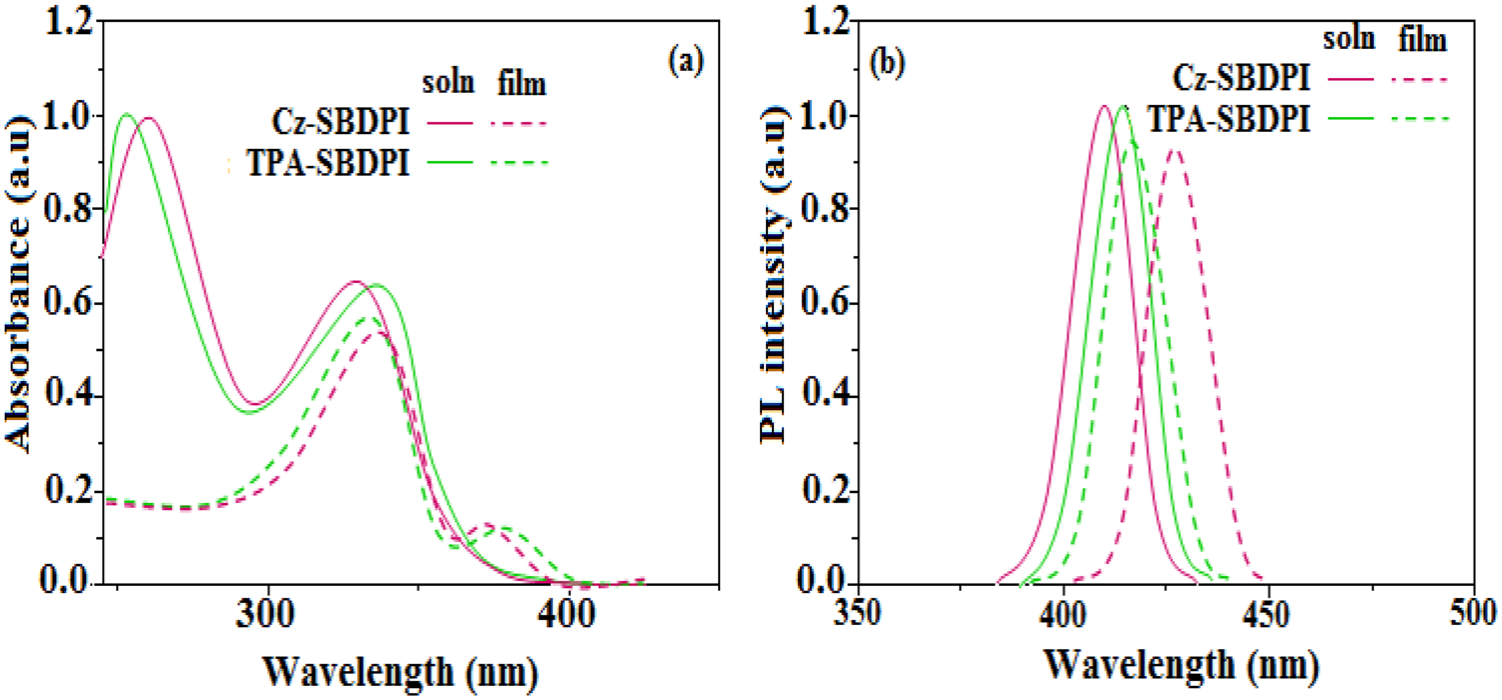
Absorption (**a**), emission (**b**) spectra of Cz-SBDPI and TPA-SBDPI phenanthrimidazoles.

Optical properties confined to electronic spectral studies of Cz-SBDPI and TPA-SBDPI properties were determined for both solution and solid state shown as Table 1 was done by recording absorption (λ_abs_) as well as emission (λ_emi_) spectra, respectively (see Fig. 4). The attachment of the nitrogen atom in phenanthrimidazole with that of the aryl group is characterized by the λ_abs_ at 262 nm, whereas λ_abs_ at 253 nm is ascribed to transition, π → π* arising from the styryl phenanthrimidazole ring and from intramolecular charge transfer that occurs between donor (carbazole/triphenylamine) to the acceptor (phenanthrimidazole), respectively^22^. Furthermore, the reason for the absorption in Cz-SBDPI and TPA-SBDPI was studied by computational methods.

Both, derivatives, Cz-SBDPI as well as TPA-SBDPI display blue emission observed at 410 and 414 nm, respectively. This phenomena is due to the absorption that results when charge transfer from donor to acceptor occurs intramolecular manner^21–23^. The developed Cz-SBDPI and TPA-SBDPI had excellent low-efficiency roll-of in a wide range of current density values. Furthermore, it was found that Cz-SBDPI and TPA-SBDPI in solution/film exhibited PLQY values of 0.80 and 0.75, respectively. It was also noticed that the blue shift was increased with higher values of molar absorptivity. The existence of strong and weak electron donors: triphenylamine and carbazole moieties is expected to enhance the efficiencies^24^. Since, core fragments in Cz-SBDPI and TPA-SBDPI are same, the newborn emitters exhibit identical absorption band. However, the EL spectra shown in Fig. 4 for Cz-SBDPI, the emission maximum was blue-shifted to 413 nm compared to TPA-SBDPI (416 nm), which induces an elongated conjugation that is stable over a wide range of applied voltages. Thus, high fluorescence efficiencies of Cz-SBDPI and TPA-SBDPI established could serve as an efficient and effective deep blue emitters. In solution, small shift to longer wavelength with respect to their corresponding film was noticed. The stacking in the solid state (films) leads to suppression of π–π* transition and accounts for this observation^25^. Similarly, the red shift of the emission peak was more pronounced with the increase in the polarity of the solvent. This variation is attributed to the changes in the polarization-induced spectral shift^26^. Hence, Cz-SBDPI show higher blue shift in both absorption and emission in comparison to TPA-SBDPI, credited to lower capability of electron donation for Cz with respect to TPA.

**Figure 5:**
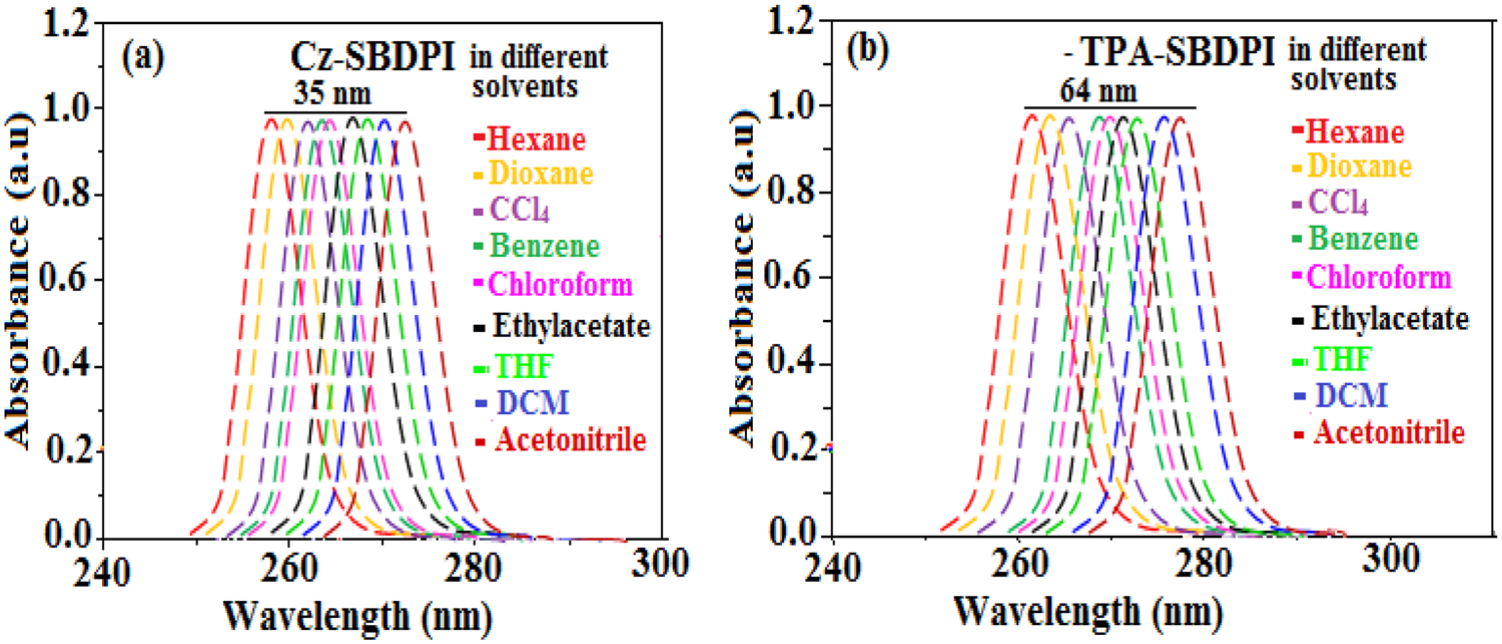
The red shift effect for (**a**) Cz-SBDPI and (**b**) TPA-SBDPI phenanthrimidazoles in different solvents monitored via UV–Vis spectra.

**Figure 6:**
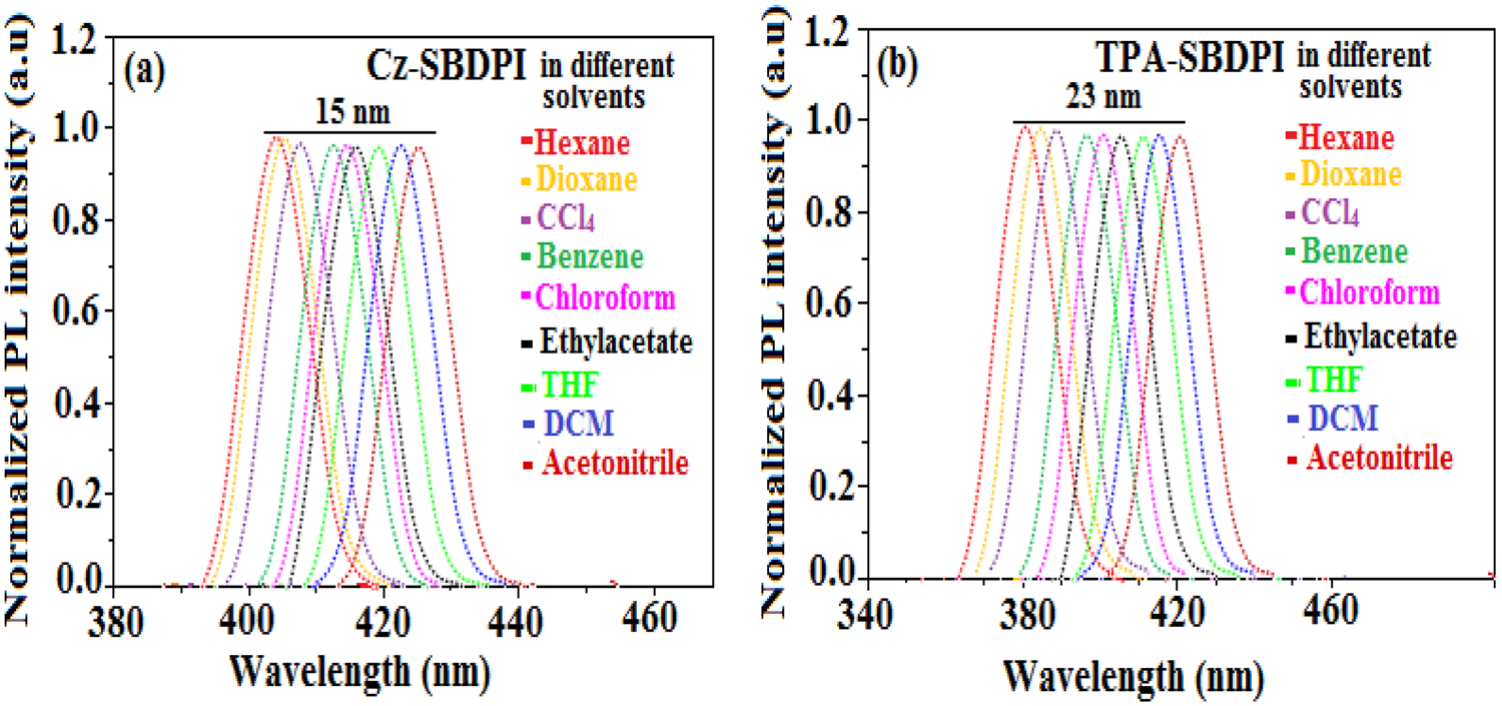
The red shift effect for (**a**) Cz-SBDPI and (**b**) TPA-SBDPI phenanthrimidazoles in different solvents observed from PL measurements.

As expected, the most probable reason that can be accounted for the blue shift is due to the reduction or suppression in CT in S_1_ emissive state with increase in LE composition. It is understood that enhancement of LE components favours red shift whereas CT reduction tends towards red shift of PL spectra. From the data obtained through experiments, it is very obvious that factor involved in CT suppression is more dominant compared to the one responsible for LE component enhancement. However, the increase of LE character results in overlapping of spectra (UV–Vis and PL) for TPA-SBDPI and Cz-SBDPI but not noticed in their parent compounds. The red shift as shown in Fig. 5 was higher in the case of TPA-SBDPI (64 nm) than Cz-SBDPI (35 nm). This observation is attributed to the solvatochromic effects that indicate the presence of CT character for excited state S_1_ at lower energy levels of TPA-SBDPI and also in Cz-SBDPI^26–28^. Likewise, in absorption bands as depicted in Fig. 6, smaller deviations of 15 and 23 nm were noted for Cz-SBDPI and TPA-SBDPI, respectively. The device's light distribution pattern was examined, and the results showed that the Lambertian factor was between 0.80 and 0.75 (Figs. S1,S2, Supporting Information). Table 1 provides a summary of all the device performances.

### HOMO and LUMO

**Figure 7:**
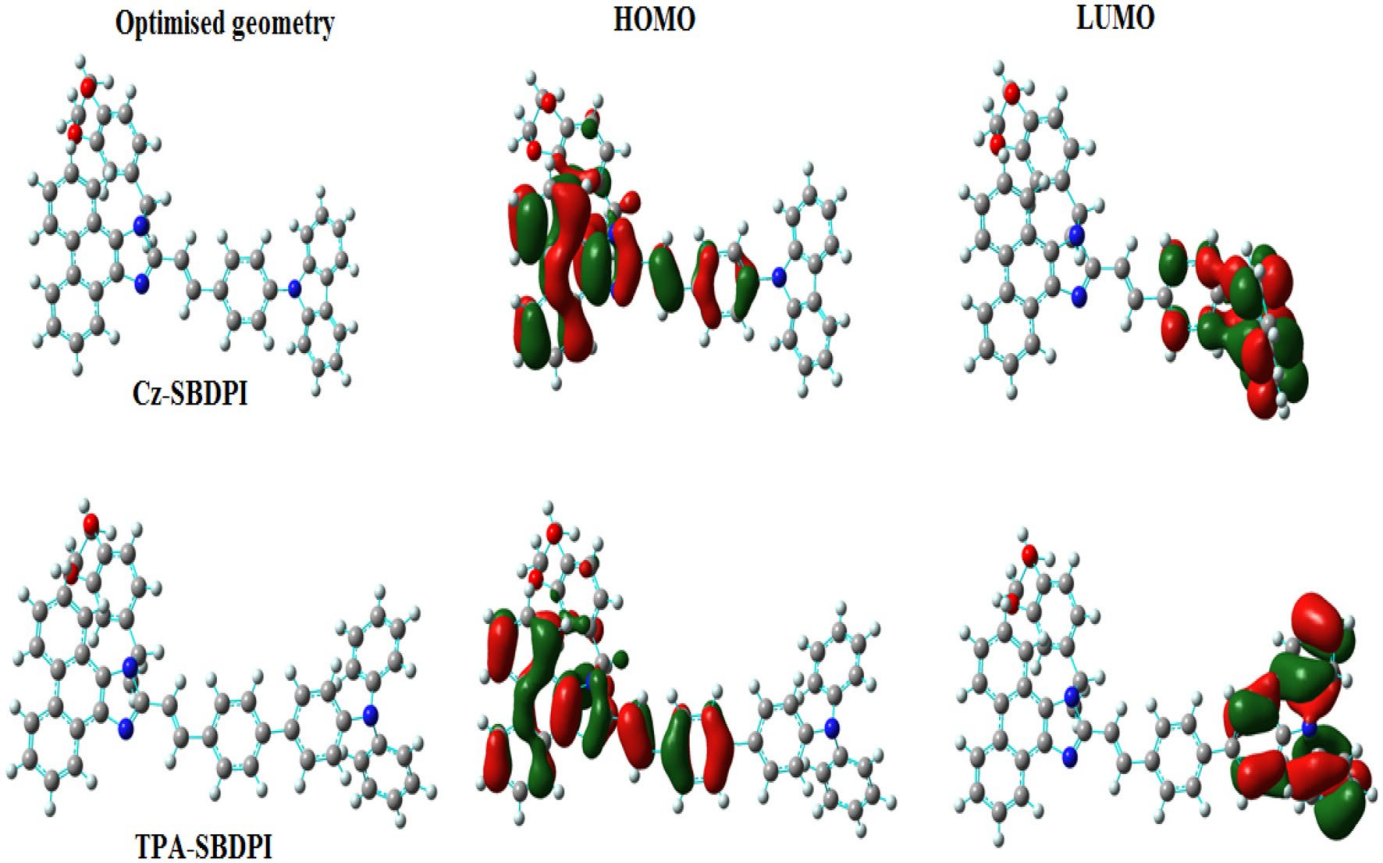
The optimized geometries along with their HOMO and LUMO for Cz-SBDPI and TPA-SBDPI phenanthrimidazoles.

**Figure 8:**
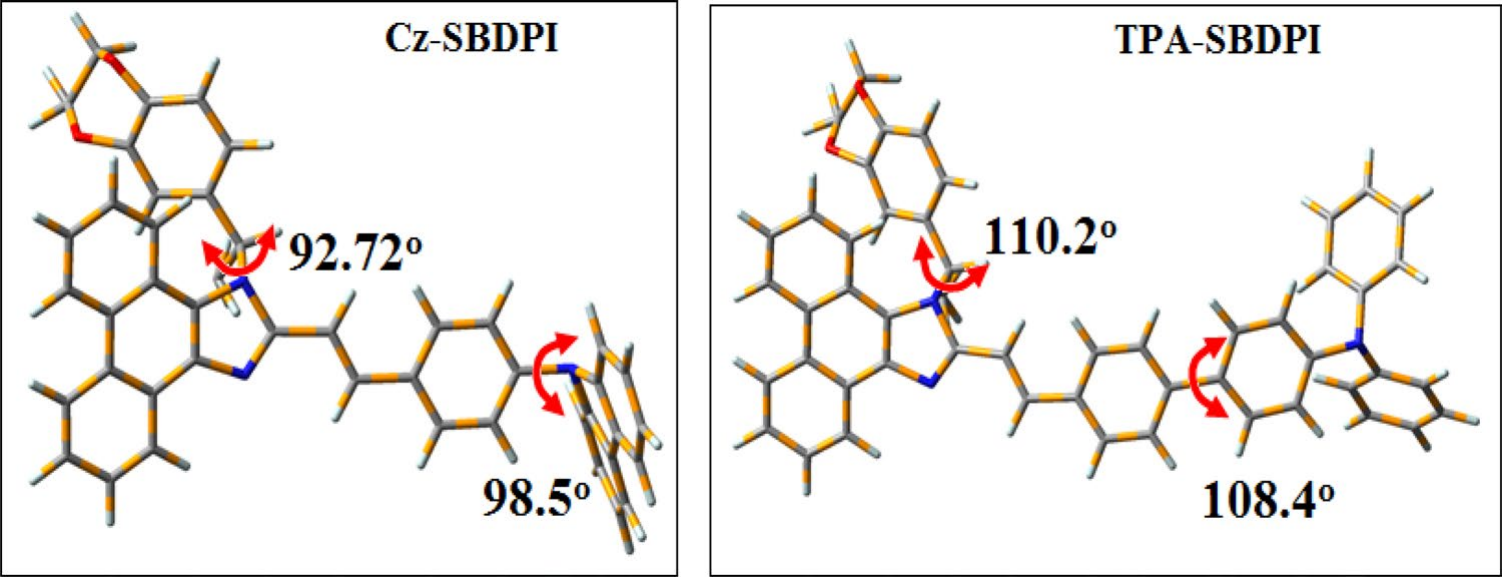
Schematic representation for dihedral angles of Cz-SBDPI and TPA-SBDPI phenanthrimidazoles.

The optimization of geometries for Cz-SBDPI and TPA-SBDPI was carried out with basis set, DFT/B3LYP/6-31G (d p) of Guassian 09 software. The optimized geometries in conjunction with their respective molecular orbital distributions, i.e., HOMO and LUMO is illustrated in Fig. 7. It was found that most of the phenanthrimidazole fragment and styryl moiety were located in HOMO orbital while Cz and TPA fragments were distributed in the LUMO orbitals. The presence of holes and adequate separation of electrons between HOMO and LUMO orbitals is responsible for the electron transport function observed in Cz-SBDPI and TPA-SBDPI. The direct value of HOMO energies of TPA-SBDPI (− 5.40 eV) and Cz-SBDPI (− 5.45 eV) were obtained from the oxidation onset potential of TPA-SBDPI (0.62 V) and Cz-SBDPI (0.58 V), respectively. This also confirm that it can act as a bipolar material. Thus, the hole and electron transport for these derivatives have to be good which is evident from its property of oxidation–reduction action. To address this issue, during calculation, simultaneous relaxation for entire geometrical parameters were done whereas the angles of torsion associated with these parameters were changed in a systematic manner from 0° to 360°. The surface energy diagram shown in Fig. 8 reveals that the minimum conformation energy corresponding to that in which the nitrogen atom (N23) of the imidazole is slanted at the angle, 92.72° (Cz-SBDPI)/110.2° (TPA-SBDPI). The attachment with the methyl substituted benzodioxin-6-amine ring and imidazole carbon atom (C25) occurs at 98.5° Cz-SBDPI)/108.4° (TPA-SBDPI) because of two types of phenyl rings present in cores of carbazole and triphenylamine, respectively. In view of the non-coplanar geometry and rigid molecular back bone, the orthogonal dihedral angles of these compounds (Cz-SBDPI and TPA-SBDPI) indicates the conformational twists for Cz-SBDPI and TPA-SBDPI^29–31^ and it is by virtue of this non-planarity of the conformation that suppresses the red shift by restricting intermolecular interaction and thereby facilitates the harvesting of high quantum efficiency (η_ex_) in films. Moreover, the presence of large moiety at C(25) in addition to side capping at N(23), provide high rigidity, increases the size which is responsible for blue emission and also enhances the thermal stabilities (*T*_*d*_ and *T*_*g*_) fulfilling all the criteria needed for efficient OLED devices (See Table 1). Also, the OLEDs lifetime are improved by high T_*d*_ and T_g_ values by forming thin films upon vacuum evaporation.

### Device performances

**Figure 9:**
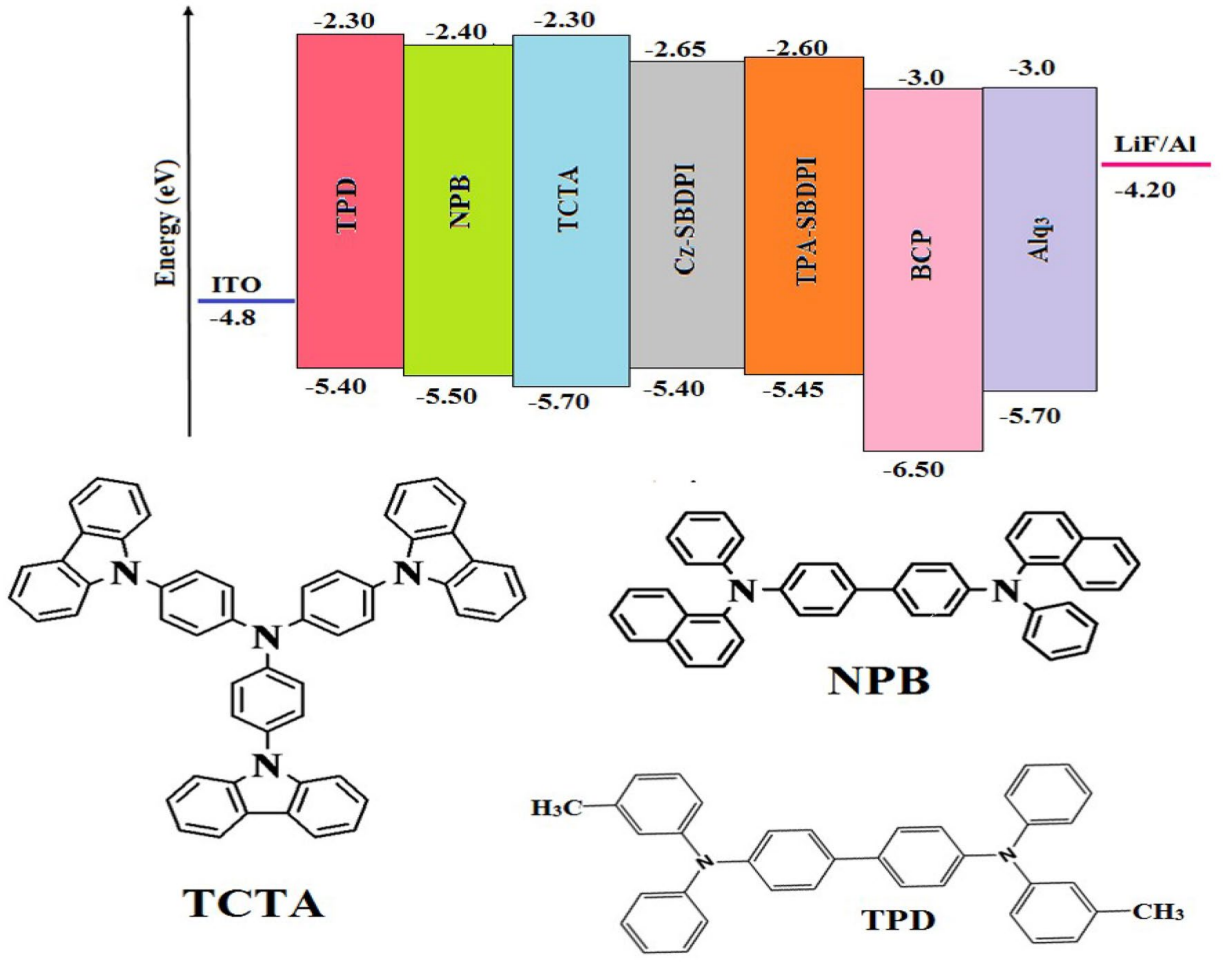
Representation of energy levels for non-doped devices of Cz-SBDPI and TPA-SBDPI phenanthrimidazoles.

**Figure 10:**
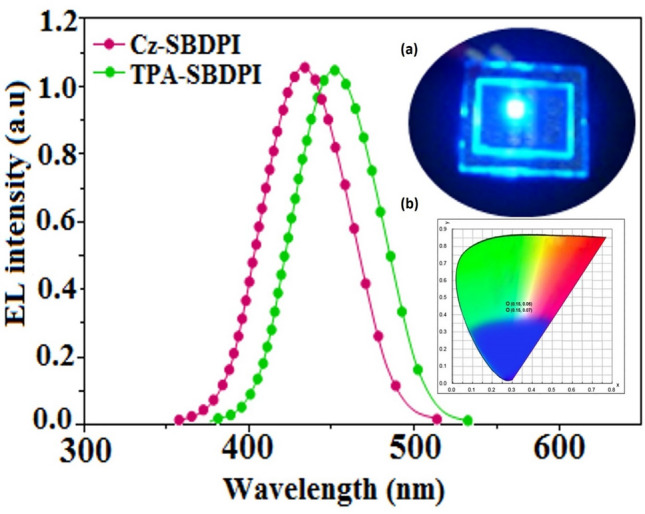
EL spectra of Cz-SBDPI and TPA-SBDPI phenanthrimidazoles. Inset: (**a**) blue material and (**b**) CIE coordinates are also provided.

The fabrication of EL device emitting blue light comprises of 3 stacked layers which are as follows: ITO/HTL (50 nm)/Cz-SBDPI/TPA-SBDPI (Emissive layers) (30 nm)/(2,9-dimethyl-4,7-diphenyl-1,10-phenanthroline) BCP (15 nm)/(tris(8-hydroxyquinolinato)aluminum) Alq3 (50 nm)/(1 nm) LiF/(100 nm) Al. Here, in this study we have suitably modified the hole transport layer with 3 different materials namely (4,40-bis[N-(1-naphthyl)-N-phenylamino]biphenyl) NPB, (N,N0-di-mtolyl-N,N0-diphenyl-1,10-biphenyl-4,40-diamine) TPD and (4,40,400-tris(N-carbazolyl)triphenylamine) TCTA to determine the EL properties of Cz-SBDPI and TPA-SBDPI phenanthrimidazoles, respectively (see Fig. 9). The similarity in both EL and PL spectra indicate that the radiative decay involved in the process is same and is due to singlet excitons (see Fig. 10). Performances of the devices were assessed and the data obtained from various factors are displayed in Table 2.

**Table 2:** Parameters associated with performance evaluation as EL device for Cz -SBDPI and TPA–SBDPI, respectively.

Emitters	Cz–SBDPI	TPA–SBDPI
V_1000_ (V)	2.5	3.1
L (cd/m^2^)	12,984	12,586
*η*_ex_ (%)	6.2	5.5
*η*_c_ (cd/A)	5.9	5.0
*η*_p_ (lm/W)	5.7	4.9
CIE (x, y)	0.15, 0.06	0.15, 0.07
EL (nm)	436	452

The wide band gap leads to increased triplet exciton lifetime which results in reduced lifetime of blue OLEDs. Developing deep blue emitters that has high efficiency and lifetime is crucial^32^. In recent past, improvements in both efficiency and lifetime for blue OLEDs has been the focus and research work is been carried out in this regard^33^. Consequently, emphasis on developing blue OLEDs that are efficient and have long-lifetime from phosphorescent and thermally activated delayed fluorescence (TADF) emitters is top priority for OLED device manufacturing industries. The device's lifetime of blue OLEDs are Cz-SBDPI 0.15, 0.06 and TPA-SBDSP 0.15, 00.7. To the very best of our knowledge, the device efficiency data of Cz-SBDPI and TPA-SBDPI and current state-of-the-art literature data for blue-emitting OLEDs reported recently have been compiled and presented as Table 3^34–46^.

**Table 3:** Summary of recent developments and state-of-art on efficiencies of blue-emitting OLEDs devices.

S. no	Emitter	EL (nm)	EQE (%)	CE (cd/A)	Von (V)	PE (lm/W)	References
1	Cz-SBDPI	436	6.20	5.90	2.50	5.70	This work
2	TPA-SBDPI	452	5.50	5.40	3.10	4.90	This work
3	Py-TPICN	440	1.34	3.00	3.60	2.62	^34–46^
4	Py-2NTF	456	1.37	2.50	3.90	1.37	^35^
5	Py-BPI	468	2.07	3.27	2.50	3.17	^36^
6	A-PI	472	0.80	1.33	3.20	0.97	^36^
7	Cz-oxa-BPh	423	4.00	1.40	3.30	1.30	^36^
8	TPE01-PPI	512	3.75	3.90	2.70	4.17	^37^
9	TPE04-PPI	508	4.07	4.22	2.70	4.51	^38^
10	TPE06-PPI	510	3.87	4.33	2.70	4.65	^38^
11	TBBA	440	3.20	2.52	3.20	2.73	^38^
12	Py-4-meTPE	472	2.50	5.14	4.00	3.05	^39^
13	Py-4mTPE	436	2.50	4.02	3.50	3.08	^40^
14	mTPE-mTEP	452	1.90	2.10	3.90	1.70	^40^
15	mTPE-pTEP	459	1.90	2.80	4.10	2.00	^41^
16	oTPE-mTEP	435	1.40	1.80	3.70	1.40	^41^
17	oTPE-pTEP	454	1.70	2.20	3.90	1.80	^41^
18	2TPE-BPI	462	3.74	4.87	2.80	5.21	^41^
19	2TPE-BPI-MCN	452	4.60	4.72	3.80	3.17	^42^
20	mTPE-2mTPE	449	1.30	1.88	4.70	1.36	^42^
21	pTPE-2mTPE	471	2.17	4.03	3.70	2.79	^43^
22	mTPE-2pTPE	461	2.09	3.67	3.50	2.90	^43^
23	CNNPI	432	2.28	1.49	3.20	1.23	^43^
24	2TPE-CNNNPI	456	2.75	3.70	3.00	2.98	^44^
25	2CP-CNNPI	454	5.09	6.65	3.00	5.66	^44^
26	Si-p-TPE	512	2.92	7.40	3.50	5.57	^44^
27	Si-tPE	488	3.38	8.04	3.00	6.17	^45^
28	Si-mTPE	432	1.21	1.39	3.70	1.18	^45^
29	PhQ-CVz	462	4.48	5.82	3.10	5.50	^45^
30	A1	434	3.62	2.84	3.67	3.02	^45^
31	A2	434	3.11	2.64	3.20	2.61	^46^
32	B1	440	3.99	3.12	4.12	3.15	^46^
33	B2	440	4.66	4.24	3.36	3.67	^46^
34	2,5-mBTPE-TP	440	1.96	3.40	4.70	1.90	^47^

**Figure 11:**
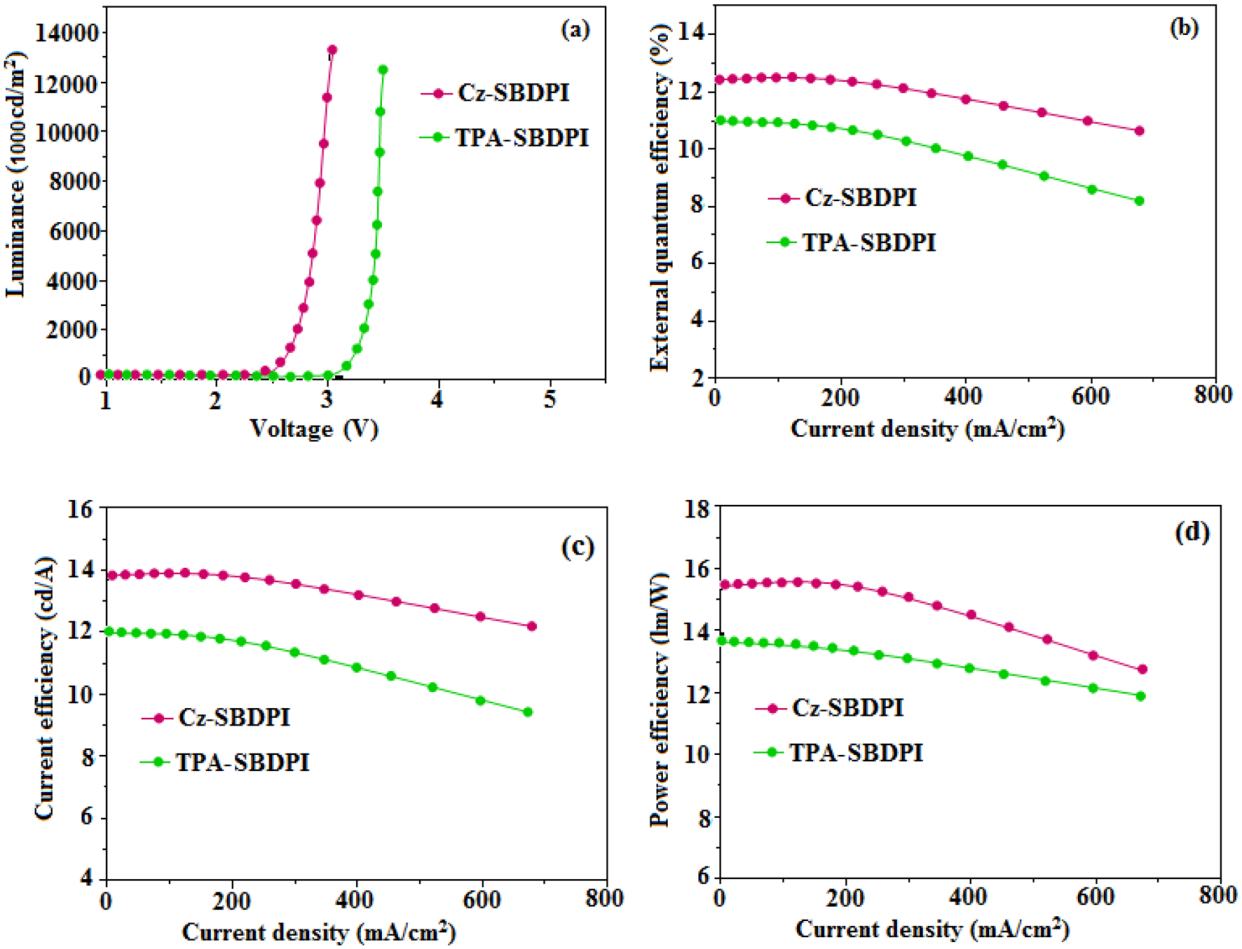
Evaluation of plots: (**a**) luminance vs. voltage; (**b**) external quantum efficiency vs. current density; (**c**) current efficiency versus current density and (**d**) power efficiency versus current density to determine the performance of Cz-SBDPI and TPA-SBDPI phenanthrimidazoles as electroluminescent materials.

In the films, both EL and PL spectra were found to be similar for Cz-SBDPI and TPA-SBDPI based devices (see Fig. 11). Among the phenanthrimidazoles, Cz-SBDPI and TPA-SBDPI based device show excellent performances. The efficiencies, both current and power for Cz-SBDPI (5.9 cd/A; 5.7 lm/W) and TPA-SBDPI (5.0 cd/A; 4.9 lm/W) devices are higher than TPA-PA (1.16 cd/A; 0.65 lm/W), TPA-NzP (1.00 cd/A; 0.77 lm/W)^47^ and *m*TPA-PPI (0.84 cd/A; 0.48 lm/W). Additionally, external quantum yield for Cz-SBDPI (80%) and TPA-SBDPI (75%) was found to be higher than (1) Cz-BzP (70%) and TPA-BzP (49.1%)^48–50^ (2) CBI (21.3%) and MCB (24.4%) and (3) PPI-pCNCz (53.8%). According to Wang et al.^51–53^ with the reduction in thickness (50–20 nm) of emissive layer, LBPPI, significant increase in current efficiencies from 0.01 to 0.67 cd/A were observed. Hence, improvements in the efficiency of these materials could be achieved by modifying the thickness of their emissive layers.

It is worthwhile to mention that the modifications in the thickness of the emissive layers to improve the efficiency and also attempts to increase the radiative rate will be the subject of investigation for our future studies. For weak donor carbazole substituted with phenanthrimidazole, the current and power efficiencies observed are 0.88 cd/A and 0.30 lm/W, respectively. The substituted carbazoles used in study performed by Gao et al*.*^54^ had values of 0.65 cd/A and 0.48 lm/W as current and power efficiencies. However, in our case, the efficiencies obtained for Cz-SBDPI were 5.9 cd/A and 5.7 lm/W, much higher than previously reported studies. Therefore, it can be concluded that both Cz-SBDPI and TPA- SBDPI are most appropriate as fluorescent based OLEDs materials.

## Conclusion

Herein, we report a simple scheme to fabricate deep blue emitting materials, viz., phenanthroimidazole derivatives (Cz-SBDPI and TPA-SBDPI) based on donor–acceptor arrangements and possess both charge-transport properties. Higher glass-transition and thermal decomposition temperatures indicate enhanced thermal stability for these compounds. The carrier transport abilities were displayed by Cz-SBDPI and TPA-SBDPI and the emission as deep blue color demonstrate that those devices that are not doped to Cz-SBDPI show maximum efficiency (*η*_ex_ − 6.2%; *η*_c_ − 5.9 cd/A; *η*_p_ − 5.7 lm/W) while efficiency is reduced for doped, TPA-SBDPI (*η*_ex_ − 5.5%; *η*_c_ − 5.0 cd/A; *η*_p_ − 4.9 lm/W). Both TPA-SBDPI and Cz-SBDPI show blue emission with CIE (0.15, 0.06) and (0.15, 0.07), respectively. The improved *η*_*s*_ for TPA-SBDPI (16.2%) and Cz-SBDPI (16%) and *η*_*IQE*_ (10.12 and 11.44%) for TPA-SBDPI and Cz-SBDPI is credited to the components involved in charge transfer that arises due to substitution of methyl groups. This work successfully devises a scheme that suitably modifies the material properties and provides a new route where bis phenanthrimidazoles with donor–acceptor molecular structures in non-doped devices and bipolar electroluminescent materials produce high performance OLED devices. Thus, the derivatives shows balanced injection/transport ability in addition to high stability that results in outstanding device performances. Hence, these carbazole donors are beneficial as they specifically generate short-wavelength based absorption or emission and are useful as blue-light-emitting materials for optoelectronic applications. The findings of the present investigation illustrate a new route to fabricate luminescent materials with donor–acceptor molecular structure that can be used to harvest efficient device performances.

### Supplementary Information

Supplementary Information.

## Data Availability

The datasets used and/or analysed during the current study available from the corresponding author on reasonable request.
