# Supplementary material for: Fabrication of high performance based deep-blue OLED with benzodioxin-6-amine-styryl-triphenylamine and carbazole hosts as electroluminescent materials
Source: Sci Rep. 2024 Jan 29;14:2432. doi: 10.1038/s41598-023-50867-x (PMC10825205; doi:10.1038/s41598-023-50867-x)
Supplement: Supplementary file 1 — Supplementary Information. [file 41598_2023_50867_MOESM1_ESM.docx]

Supplementary Materials:

# Manuscript ID: 2d198db4-1299-4359-893e-6b3854e00f53 (REVISED)

**Title of the Manuscript: “Fabrication of High Performance based deep-blue OLED with Benzodioxin-6-amine-styryl-triphenylamine and Carbazole hosts as Electroluminescent Materials”**


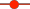

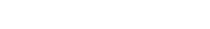

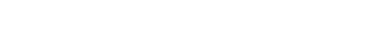

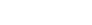

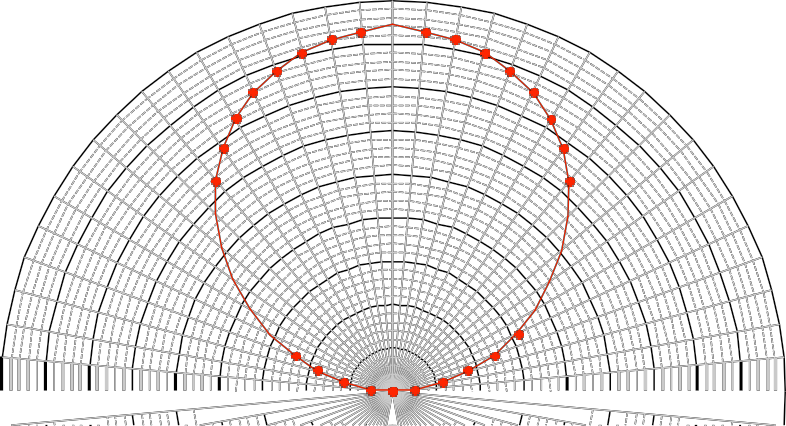

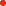

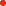

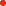

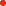

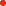

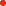

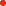

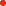

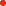

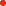

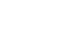

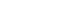

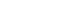

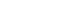

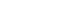

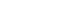

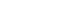

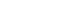

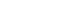

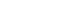

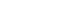

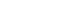

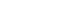

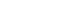

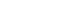

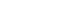

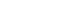

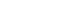

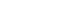

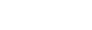

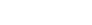

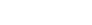

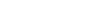

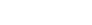

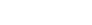

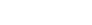

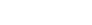

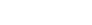

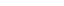


Unit Solid Angle

Unit Solid Angle for Lambertian

**0.09 0 5 10**

**15**

**20**

**0.08**

**25**

**30**

**35**

**0.07**

**40**

**45**

**0.06**

**0.05**

**50**

**55**

**0.04**

**0.03**

**0.02**

**0.01**

**60**

**65**

**70**

**75**

**80**

**85**

**90**

**0.00**

**Figure S1.** The angular dependence of luminous intensity (Lambertian factor: 0.80)


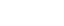

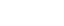

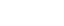

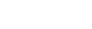

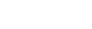

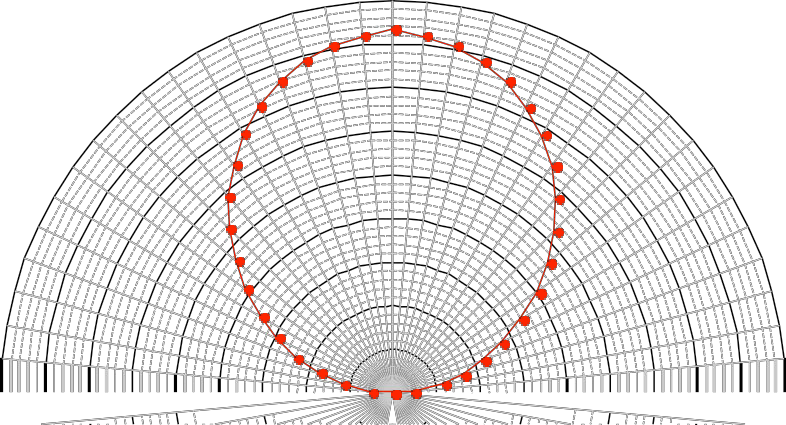

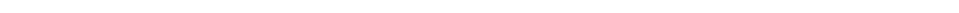

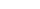

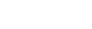

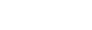

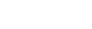

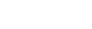

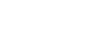

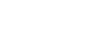

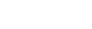

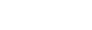

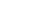

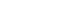

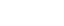

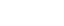

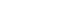

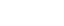

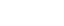

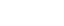

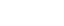

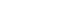

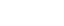

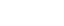

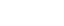

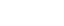

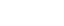

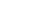

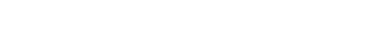

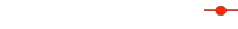


Unit Solid Angle

Unit Solid Angle for Lambertian

**0.09 0 5**

**10**

**15**

**20**

**0.08**

**25**

**30**

**35**

**0.07**

**40**

**45**

**0.06**

**50**

**0.05**

**0.04**

**0.03**

**0.02**

**0.01**

**55**

**60**

**65**

**70**

**75**

**80**

**85**

**90**

**0.00**

**Figure S2.** The angular dependence of luminous intensity (Lambertian factor: 0.75)

The above figures are angular dependence of luminous intensity of fabricated high performance based deep-blue OLED materials.
